# Study on Calculation Method of Bending Performance of Concrete Sandwich Composite Slab

**DOI:** 10.3390/ma17112591

**Published:** 2024-05-28

**Authors:** Mai-Li Cheng, Guo-Zhuang Hu, Hong-Qi Wang

**Affiliations:** 1 School of Architectural Engineering, Yanan University, Yan’an 716000, China; 13619526551@163.com; 2 SCEGC NO.13 Construction Engineering Group Co., Ltd., Yan’an 716000, China; wanghongqi001@126.com

**Keywords:** concrete sandwich panel, concentrated support, differential equation, bending performance, calculation method

## Abstract

In order to explore the flexural behavior of a concrete sandwich panel under concentrated boundary conditions, based on Kirachhoff’s elastic thin plate theory in this paper, the geometric deformation, physical conditions, and equilibrium relationship of a sandwich panel are deduced by constructing the layered analysis model of the sandwich panel, the basic differential equation of the flexural deformation of the concrete sandwich thin plate is obtained, and the mathematical expression of the internal force and displacement under the boundary condition of concentrated support is given. Combined with an engineering example, the proposed calculation method is verified. The results show that, in the arrangement of reliable connectors for concrete sandwich panels, the concrete wythes bear the load while the contribution of the core layer to the bending capacity of the structure can be ignored. When subjected to a laterally distributed load, the sandwich panel mainly experiences out-of-plane bending deformation, and the bending normal stress in the concrete panel layer shows a linear non-uniform distribution along the thickness direction of the panel. The bending deformation performance and bearing efficiency of a concrete sandwich slab with the change in concentrated support position have significant effects, and the load transfer efficiency can be improved by optimizing the arrangement of supports. Except for small local areas near the supports, the bending stress distribution and deformation behavior of the concrete sandwich panel can be accurately analyzed by the calculation method established in this paper.

## 1. Introduction

Concrete sandwich panels consist of high-strength concrete surface layers and lightweight soft core layers. This type of structure is characterized by its lightweight, high strength, high rigidity, thermal insulation (sound insulation, vibration isolation), and other features. It is extensively utilized in prefabricated building external walls, floors, and roof panels [1,2]. However, significant variations in the mechanical properties among the layers of sandwich panels, along with the complex operating mechanisms of composite panels, present substantial challenges for the precise design and implementation of this type of sandwich composite building material.

To achieve the dual enhancement of the load-bearing capacity and thermal insulation performance in concrete sandwich panels, a multitude of theoretical, experimental, and numerical simulation studies have been carried out globally on concrete sandwich composite panel structures. The research on concrete sandwich panel structures predominantly encompasses the material properties of the wythe layer and core layer, the material and form of the connectors, the construction of the sandwich structures, and the mechanical load-bearing performance of the components. Table 1 provides an overview of the current research status in this field.

**Figure 1:**
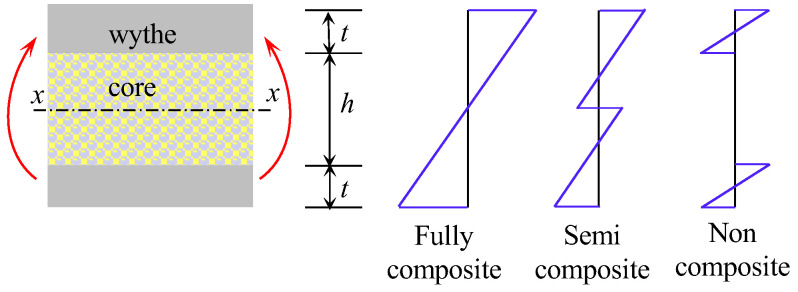
Sandwich panel load-bearing mode schematic.

The thickness of the concrete panels cannot be ignored in structural analysis, as traditional sandwich panel theories and some assumptions are no longer applicable [27]. References [8,21,25] studied the bending capacity and failure modes of concrete sandwich panels through model tests and finite element simulations, and established some theoretical analysis models and calculation formulae for flexural deflection. Concrete sandwich panels exhibit significant differences in bending load-bearing modes under the lateral loads corresponding to different connector performances. The load-bearing modes include fully composite, semi-composite, and non-composite action [18,28]. The schematic diagram of bending load-bearing modes of sandwich panels is shown in Figure 1. The American PCI Manual does not provide design methods for internal forces and deformations of partial composite action [29], while the current Chinese design code recommends calculation using finite element methods [30]. Enhancing the bending load-bearing capacity of concrete sandwich panels often involves increasing the design of connectors between the wythes to enable the panels to exhibit fully or semi-composite action modes.

Connectors play a crucial role in the joint force transmission of concrete sandwich panels, with their structure and performance significantly influencing the load-bearing capacity of sandwich panels [31]. With the increasing research on concrete sandwich panel structures, various forms of connectors for sandwich panels have emerged, including Z-reinforced (BFRP [7] and GFRP [19]), space fabric net [15], reinforced truss [12], steel wire truss [18], concrete cylinders [17], steel wire mesh [21], hollow glass FRP tubes [8], short UHPC columns [25], concrete ribs [9], and rivets [26]. Different forms of connectors have a significant influence on the composite action of concrete wythes.

The bending load-carrying mechanism of concrete sandwich panel structures mainly relies on the high-strength wythe layer to bear and transfer loads; thus, the mechanical properties of the wythe layer material are crucial for the load-carrying capacity of sandwich panel structures. Currently, wythe layer materials used in the construction industry for sandwich panel structures include foam concrete [22], steel wire concrete [18], RC [8], CFRP [24], TRC [16], glass plain-weave fabric [23], RA concrete [20], steel fiber concrete [19], and fine stone concrete [21]. By selecting appropriate wythe layer materials, the load-carrying capacity and performance of sandwich panel structures can be significantly improved. However, for the core layer materials, lightweight and thermally insulating functional materials are mainly selected, including EPS [11], XPS [14], EPS mortar [16], PVC foam [23], and foam concrete [15].

The internal force carries properties of different components in building structures. Wall components primarily withstand axial or eccentric pressure, whereas roof and floor components mainly support bending loads. References [9,11,14,22,32] studied the bending performance of concrete sandwich panel structures under transverse loads, mainly investigating cracking patterns, strength, stiffness, and other indicators. Reference [33] discussed the bending load-bearing capacity by conducting bidirectional plate bending experiments, while reference [7] studied the out-of-plane bending behavior of wall panels. The compressive performance of concrete sandwich panels was the main focus of the study in references [3,5], investigating the load-carrying modes of sandwich wallboard structures under axial pressure and eccentric pressure. As for the shear performance of concrete sandwich panels, reference [15] studied the shear carrying capacity of TRC panels. In addition, references [4] and [10] studied the seismic performance of concrete sandwich panel structures through shaking table tests and pseudo-static cyclic tests, while reference [24] mainly investigated the vibrational behavior of CFRP foam panels. Reference [16] discussed the bending capacity and failure modes of fiber fabric-reinforced concrete sandwich panels (TRC) under dynamic impact. References [13] and [34] conducted a theoretical analysis and numerical simulation on the effects of concrete sandwich components under temperature effects. References [35] and [12] explored the stress characteristics of arch-shaped and flat-shaped concrete sandwich panel structures at different construction stages, providing analysis models and methods. Reference [6] examined long-span concrete sandwich panel structures by constructing steel-concrete composite arch-shaped sandwich roofs.

Based on a review analysis of concrete sandwich panel structures, it is evident that the stress distribution along the thickness direction of the concrete wythe layer in sandwich structures is uneven. Furthermore, there are few reported theoretical models for the bending analysis of concrete sandwich panels under concentrated support boundary conditions.

Therefore, this study focuses on the concrete sandwich panel structure under concentrated support, using Kirchhoff’s theory of thin plate elasticity to investigate the bending performance and calculation methods of sandwich panels. By establishing geometric, physical, and equilibrium conditions, the differential equations for the deflection deformation of sandwich panels are obtained, and validated through engineering examples. Additionally, the study investigates the load transfer efficiency and deformation patterns of concrete sandwich panels under concentrated support boundary conditions.

## 2. Concrete Sandwich Panel Calculation Model

The concrete sandwich panel structure will exhibit a bending stress state under the action of lateral loads within the plane. Based on Kirchhoff’s plate theory of elasticity and considering the bending capacity mechanism and deformation characteristics of concrete sandwich panels under a lateral load, an out-of-plane bending theoretical calculation model for concrete sandwich panels is developed.

### 2.1. Calculation Assumptions

The upper and lower wythes of concrete sandwich panels are typically connected using connectors such as steel wire mesh and steel reinforcement trusses, forming reliable connections that create a good joint action between the wythes. The theoretical model assumes the following [27]:

(1) The layer of the sandwich panel is uniform in material and continuous in the plane, considered as an ideal elastic body;
(2) Under loading, the flexural deformation of the sandwich panel is much smaller compared to the thickness, assuming the displacement and deformation of the sandwich panel are small;
(3) During bending deformation, the sandwich panel does not undergo in-plane deformation, and the mid-surface normal remains straight before and after deformation;
(4) During bending deformation, there is no compressive stress or deformation in each layer of the sandwich panel, *σ_z_* = 0, *ε_z_* = 0;
(5) The material of the core layer is soft and has low stiffness. The stress components of the core layer in the *XOY* plane are neglected, and does not bear internal forces in the structure, σ*_x_* = *σ_y_* = *τ_xy_* = 0.

### 2.2. Sandwich Panel Element Analysis

**Figure 2:**
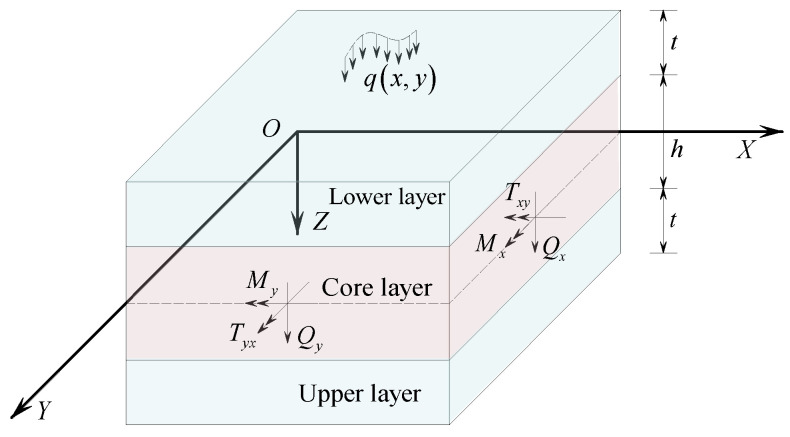
Concrete sandwich panel analysis model.

Establishing an *XOY* plane at the mid-surface of the concrete sandwich panel, with the *Z*-axis pointing downwards as positive, the upper and lower layers of the sandwich panel are concrete panels oriented in the same and opposite directions to the *Z*-axis. Both upper and lower layers have a thickness of *t*, while the core layer has a thickness of *h*. A distributed load *q*(*x*, *y*) acts on the lower surface of the sandwich panel along the positive *Z*-axis. Figure 2 illustrates a schematic diagram of the sandwich panel analysis.

#### 2.2.1. Geometric Conditions

**Figure 3:**
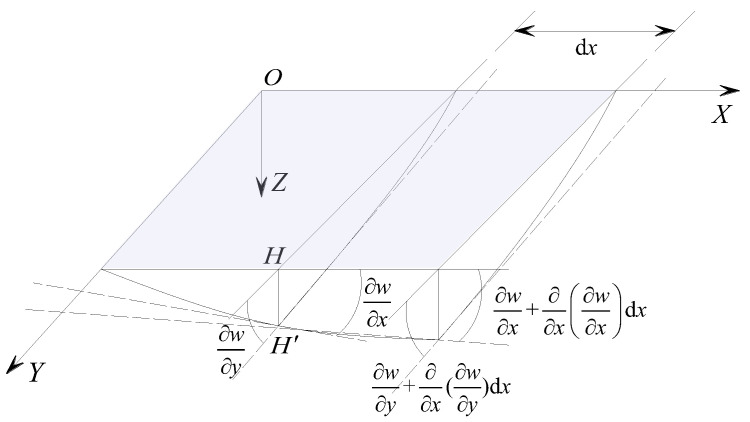
Mid-surface bending analysis.

Due to the significantly larger bending deformation compared to shear deformation in the sandwich panel, in the analysis of overall deformation of the concrete sandwich panel, based on assumptions (1)~(3), only the bending deformation of the mid-surface of the sandwich panel can be considered for analysis. The calculation schematic is shown in Figure 3.

When the sandwich panel is subjected to lateral loading, the perpendicular deflection of point *H* on the mid-surface of the panel is *w*(*x*, *y*). The tangential slopes in the *X*-axis and *Y*-axis directions at point *H*′ after deformation can be expressed as:

(1) $$\left\{\begin{array}{c} \varphi_{x}=\frac{\partial w}{\partial x}\\ \varphi_{y}=\frac{\partial w}{\partial y} \end{array}\right.$$

Combining the small deformation basic assumption (2), the curvature of the deflection curve along the *X*-axis and *Y*-axis directions at point *H*’ can be approximately expressed as:

(2) $$\left\{\begin{array}{c} \kappa_{x}\approx -\frac{\partial^{2}w}{\partial x^{2}}\\ \kappa_{y}\approx -\frac{\partial^{2}w}{\partial y^{2}} \end{array}\right.$$

The deflection surface of the plate is a function of the co-ordinates *x* and *y*; therefore, the torsion rates of the slope of the deformation curve along the *X*-axis and *Y*-axis directions on the plate can be expressed as:

(3) $$\kappa_{xy}=-\frac{\partial^{2}w}{\partial x\partial y}$$

#### 2.2.2. Physical Conditions

Due to significant variations in the mechanical properties of the materials within concrete sandwich panels, the analysis typically assumes that the core layer and wythe layer share the responsibility for withstanding shear forces, while the concrete face panel layer primarily supports bending moments. Separate calculation models can be established for the core layer and the concrete face panel layer [12].

(1) Core Layer

**Figure 4:**
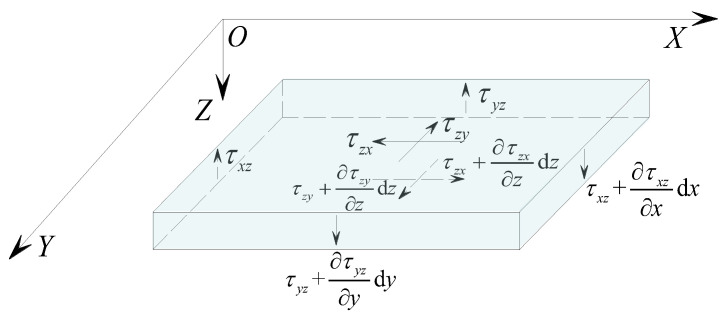
Analysis of stress in the core layer.

Based on assumptions (4) and (5), Figure 4 shows the stress relationship of the core layer micro-elemental. It can be determined from the equilibrium conditions of the micro-element that

(4) $$\left\{\begin{array}{c} \frac{\partial \tau_{xz}}{\partial z}=0\\ \frac{\partial \tau_{yz}}{\partial z}=0 \end{array}\right.$$

This indicates that *τ_xz_* and *τ_yz_* are functions of *x* and *y*, and shear stress is uniformly distributed along the thickness of the core layer.

Let *Q_x_* and *Q_y_* be the total lateral shear forces of the core panel; then,

(5) $$\left\{\begin{array}{c} \tau_{xz}=\frac{Q_{x}}{h+2t}\\ \tau_{yz}=\frac{Q_{y}}{h+2t} \end{array}\right.$$

According to the two-dimensional Hooke’s Law, the corresponding shear strain is

(6) $$\left\{\begin{array}{c} \gamma_{xz}=\frac{\partial w}{\partial x}+\frac{\partial u}{\partial z}=\frac{Q_{x}}{G_{e}\left(h+2t\right)}\\ \gamma_{yz}=\frac{\partial w}{\partial y}+\frac{\partial v}{\partial z}=\frac{Q_{y}}{G_{e}\left(h+2t\right)} \end{array}\right.$$

where *G_e_* is the equivalent shear modulus of the concrete core panel, taken as $G_{e}=\frac{h\times G_{c}+2t\times G_{s}}{h+2t}$, and *G_c_* and *G_s_* are the shear modulus of the core layer and the concrete face panel layer, respectively.

Considering the deformation displacements *u*, *v*, and *w* of the sandwich panel in the *X*-axis, *Y*-axis, and *Z*-axis directions, by introducing the parameter of the flexural curvature of the sandwich panel, combined with assumption (3), the transverse shear force of the sandwich panel is given by

(7) $$\left\{\begin{array}{c} Q_{x}=G_{e}\left(h+2t\right)\left(\frac{\partial w}{\partial x}+\frac{\partial u}{\partial z}\right)\\ Q_{y}=G_{e}\left(h+2t\right)\left(\frac{\partial w}{\partial y}+\frac{\partial v}{\partial z}\right) \end{array}\right.$$

(2) Concrete Panel Layer

**Figure 5:**
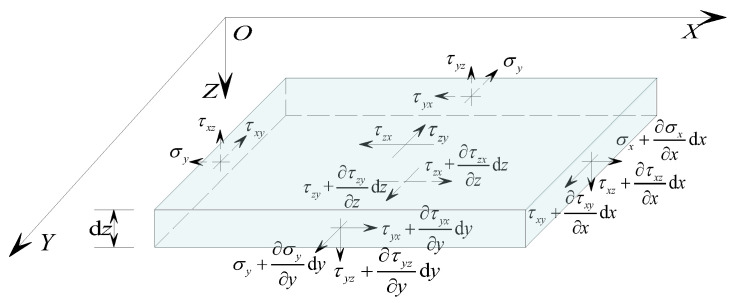
Stress analysis of concrete panels.

The stress micro-element of the concrete panel layer is as shown in Figure 5. Assuming the displacements of the mid-surface in the *X*-axis and *Y*-axis directions of the upper layer of the sandwich panel are *u*^T^ and *v*^T^, the displacement of the mid-surface of the upper layer can be expressed as:

(8) $$\left\{\begin{array}{c} u^{T}=\frac{h+t}{2}\varphi_{x}\\ v^{T}=\frac{h+t}{2}\varphi_{y} \end{array}\right.$$

According to Hooke’s Law, the normal stress in the mid-surface of the upper layer is

(9) $$\left\{\begin{array}{c} \sigma_{x}^{T}=\frac{E_{s}\left(h+t\right)}{2\left(1-\mu_{s}^{2}\right)}\left(\kappa_{x}+\mu \kappa_{y}\right)\\ \sigma_{y}^{T}=\frac{E_{s}\left(h+t\right)}{2\left(1-\mu_{s}^{2}\right)}\left(\kappa_{y}+\mu \kappa_{x}\right)\\ \tau_{xy}^{T}=\tau_{yx}^{T}=\frac{E_{s}(h+t)}{2(1+\mu_{s})}\kappa_{xy} \end{array}\right.$$

where *E_s_* and *μ_s_* represent the elastic modulus and Poisson’s ratio of the panel layer concrete.

According to assumption (3), due to the linear distribution of bending tensile stress along the thickness direction of the upper layer, making $\sigma_{x,u}^{T},\;\;\sigma_{y,u}^{T},$ and $\tau_{xy,u}^{T}$ as the tensile stress and shear stress on the upper surface of the upper layer, and $\sigma_{x,l}^{T},\;\;\sigma_{y,l}^{T},$ and $\tau_{xy,l}^{T}$ as the tensile stress and shear stress on the lower surface of the upper layer, then

(10) $$\left\{\begin{array}{c} \sigma_{x,u}^{T}=\frac{h+2t}{h+t}\cdot \sigma_{x}^{T},\sigma_{y,u}^{T}=\frac{h+2t}{h+t}\cdot \sigma_{y}^{T},\tau_{xy,u}^{T}=\tau_{yx,u}^{T}=\frac{h+2t}{h+t}\cdot \tau_{xy}^{T}\\ \sigma_{x,l}^{T}=\frac{h}{h+t}\cdot \sigma_{x}^{T},\sigma_{y,l}^{T}=\frac{h}{h+t}\cdot \sigma_{y}^{T},\;\tau_{xy,l}^{T}=\tau_{yx,l}^{T}=\frac{h}{h+t}\cdot \tau_{xy}^{T} \end{array}\right.$$

Due to the linear distribution of bending tensile stress, the stress in the mid-layer of the lower panel is equal in magnitude but opposite in sign to the upper layer.

The total bending moment (*M_x_, M_y_*) and total torsional moment (*T_xy_* = *T_yx_*) of the sandwich panel can be calculated using the equivalent stresses in the mid-layer of the upper and lower panels:

(11) $$\left\{\begin{array}{c} M_{x}=(h+t)t\cdot \sigma_{x}^{T}\\ M_{y}=(h+t)t\cdot \sigma_{y}^{T}\\ T_{xy}=T_{yx}=(h+t)t\cdot \tau_{xy}^{T} \end{array}\right.$$

Substituting Equation (9) into Equation (11) yields the physical conditions of the concrete sandwich panel:

(12) $$\left\{\begin{array}{c} M_{x}=-\frac{E_{s}{\left(h+t\right)}^{2}t}{2\left(1-\mu_{s}^{2}\right)}\cdot \left(\kappa_{x}+\mu \kappa_{y}\right)\\ M_{y}=-\frac{E_{s}{\left(h+t\right)}^{2}t}{2\left(1-\mu_{s}^{2}\right)}\cdot \left(\kappa_{y}+\mu \kappa_{x}\right)\\ T_{xy}=T_{yx}=-\frac{E_{s}{\left(h+t\right)}^{2}t}{4\left(1+\mu_{s}\right)}\cdot \kappa_{xy} \end{array}\right.$$

#### 2.2.3. Equilibrium Conditions

**Figure 6:**
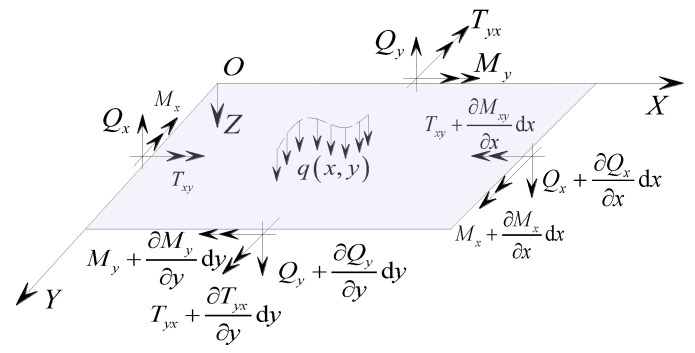
Force equilibrium analysis.

According to assumption (2), the influence of the deformation displacement of the sandwich panel on the internal forces of the structure is negligible, and geometric non-linearity effects are not considered. By considering the equilibrium force system of the sandwich panel as shown in Figure 6, the equilibrium condition of the micro-element is as follows:

(13) $$\left\{\begin{array}{c} \sum F_{x}=0,\;\frac{\partial Q_{x}}{\partial x}+\frac{\partial Q_{y}}{\partial y}+q=0\\ \sum M_{Y}=0,\;\frac{\partial M_{x}}{\partial x}+\frac{\partial T_{yx}}{\partial y}-Q_{x}=0\\ \sum M_{X}=0,\;\frac{\partial M_{y}}{\partial y}+\frac{\partial T_{xy}}{\partial x}-Q_{y}=0 \end{array}\right.$$

where *q* represents the transverse load distribution intensity of the sandwich panel, with the direction defined as positive with the *Z*-axis, and negative when in the opposite direction.

In Equation (13), the three equilibrium equations for the sandwich panel involve five basic unknowns. Introducing geometric and physical conditions is necessary for the establishment of the fundamental differential equations for solving displacements. By eliminating *q_x_* and *q_y_* simultaneously from Equation (13) and incorporating Equation (12), the fundamental differential equation for the small deflection bending of the sandwich panel can be derived as:

(14) $$\frac{\partial^{4}w}{\partial x^{4}}+(1+\mu_{s})\frac{\partial^{4}w}{\partial x^{2}\partial y^{2}}+\frac{\partial^{4}w}{\partial y^{4}}=\frac{2(1-\mu_{s}^{2})}{E_{s}{\left(h+t\right)}^{2}}\cdot q(x,y)$$

## 3. Concentrated Support Boundary Conditions

**Figure 7:**
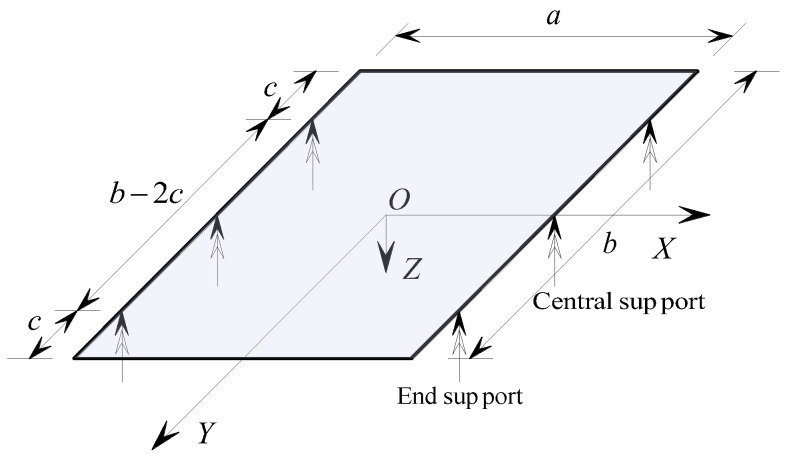
Simplified diagram for the calculation of a concentrated-supported hinge boundary (unit: mm).

When solving the fundamental differential equations for the bending of concrete sandwich panels, it is necessary to ensure that the particular solutions of the differential equations satisfy the specified boundary conditions. Referring to the simplified analysis diagram of the concrete sandwich panel supported by a concentrated hinge support at a single point as depicted in Figure 7, the concentrated support boundary conditions for the concrete sandwich panel can be divided into internal force boundaries and displacement boundaries, with a coupling between the internal force and displacement boundaries.

### 3.1. Internal Force Boundary

For each concentrated-supported hinge boundary on the plate, there are no bending constraints. Therefore, the corresponding boundary bending moment should be zero. That is, when $x=\pm \frac{a}{2}$, the total bending moment *M_x_* on the boundary of the sandwich plate parallel to the *Y*-axis is zero, indicating $\frac{\partial^{2}w}{\partial x^{2}}=0$; when $y=\pm \frac{b}{2}$, the total bending moment *M_y_* on the boundary of the sandwich plate parallel to the *X*-axis is zero, indicating $\frac{\partial^{2}w}{\partial y^{2}}=0$.

For concentrated fixed-supported boundaries, the bending moment internal forces are zero for the free boundary sections except at the supported points.

### 3.2. Displacement Boundary

At the concentrated-supported hinge boundaries of the sandwich panel, the vertical displacement at the support points is zero. That is, for the support boundary $x=\pm \frac{a}{2}$, when $y=\pm (\frac{b}{2}-c)$ and *y* = 0, the vertical displacement of the sandwich panel *w* = 0; when there is no support at *y* = 0, the boundary degenerates into a four-point support; when *c* = 0, the support conditions degenerate into a corner support.

For concentrated fixed support boundaries, each support point constrains the translational and rotational displacements of the panels, and additional conditions of zero rotation and displacement at the support points need to be supplemented based on the above hinged support boundaries.

## 4. Engineering Example

To further validate the proposed calculation model in this paper, a simplified rectangular concrete sandwich roof structure was designed based on the structural characteristics of concrete sandwich panels. The concrete sandwich panel is supported by four-point concentrated hinged supports. The concrete sandwich panel has plan dimensions of 4000 mm × 8000 mm and a design thickness of 200 mm. The thickness of the upper and lower concrete wythe layers of the sandwich panel is both 60 mm, and the strength Grade C30. A single-layer bidirectional steel mesh is placed within the concrete wythe, with both the longitudinal and transverse steel bar spacing at 400 mm. The steel mesh in the upper and lower panels is connected by truss steel bars, with a diameter of 10 mm and a strength grade of HRB335. The core layer is made of XPS with a thickness of 80 mm. The material mechanical properties of concrete, XPS, and reinforcement are shown in Table 2. A uniform transverse surface load of *q* = 2.0 kN·m^−2^ is applied on the roof surface of the concrete wythe [36]. The study analyzed the deformation characteristics of the sandwich panel when subjected to concentrated hinge support displacement along the *Y*-direction.

**Figure 8:**
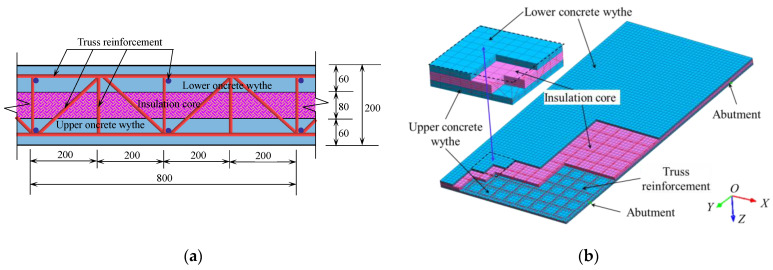
Sectional dimensions and finite element model of concrete sandwich panel: (**a**) cross-sectional structure and dimensions (unit: mm); and (**b**) finite element model.

**Figure 9:**
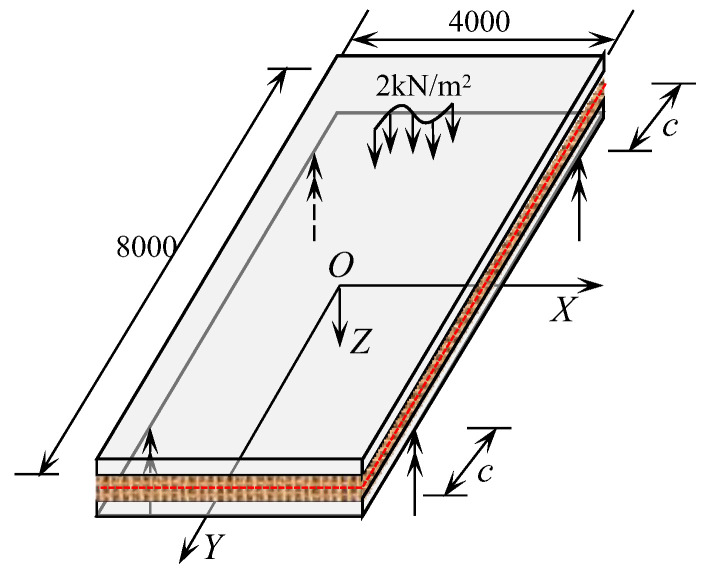
Schematic diagram of the calculation model for rectangular panels (unit: mm).

With the aid of finite element analysis software Midas Fea NX (Beijing MIDAS Technology Co., Ltd., Beijing, China), a finite element model of the engineering example is established. The wythe and core layers are modeled using 3D hexahedral elements, with a total number of 38,400, and the reinforcement is modeled using 1D linear embedded truss elements, with a total number of 4961. The construction of the concrete sandwich panel and partial cross-sectional dimensions are shown in Figure 8a, and the established finite element analysis model is shown in Figure 8b. According to the calculation method of the concrete sandwich panel established in this paper, the differential equations for the small deflection of the thin core plate are solved, and the results are verified by combining them with the finite element analysis results. The schematic of the structural calculation model of the sandwich panel is shown in Figure 9.

## 5. Results Analysis and Discussion

By analyzing the flexural deformation and bending moment internal force characteristics of concrete sandwich panels under four-point support boundaries, the study investigates the load transfer characteristics of concrete sandwich panel structures with concentrated hinged support.

### 5.1. Verification of Calculation Methods

#### 5.1.1. Stress Analysis

**Figure 10:**
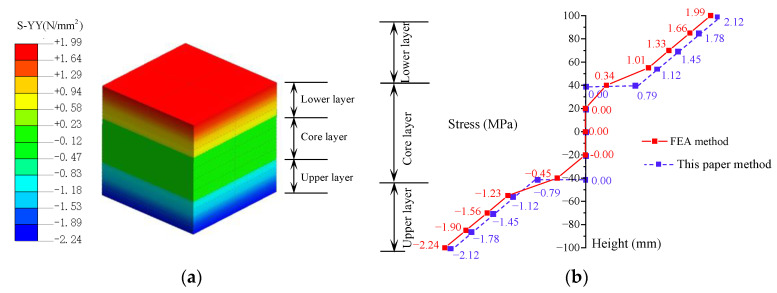
Bending stress distribution of the sandwich panel: (**a**) *σ_yy_* stress distribution; and (**b**) bending stress distribution model.

Under transverse loading, concrete sandwich panels primarily experience bending stresses, while significant shear stresses are present in the localized support areas. This study focuses on the analysis of bending stresses in the core region of the sandwich panel (*x* = −100~100 mm, *y* = −100~100 mm). Based on the theoretical model established in this paper for sandwich panels and finite element analysis results, Figure 10 shows the distribution of *Y*-direction bending tensile stress (*σ_yy_*) in the central region of the concrete sandwich panels under the condition of support end distance *c* = 0 mm.

From Figure 10, it can be observed that, under transverse loading, the bending tensile stress in concrete sandwich panels shows a generally linear distribution along the thickness of the panel. Due to the relatively small elastic modulus of the core layer polystyrene material, the bending stress in the core layer is minimal under the assumption of no slip between the wythe layer and the core layer. In practical engineering applications, due to significant differences in the mechanical properties of the materials in the various layers of the sandwich panel and the non-absolute rigidity of the vertical connections between the layers, the distribution of bending stresses under transverse loading is complex (red solid line). For simplified calculation purposes, this study simplifies the bending stress in the sandwich panel to be linearly distributed in the concrete panel layer and zero in the core layer (blue dashed line). A comparison shows that the bending stress distribution pattern of concrete sandwich panels obtained by the method in this study is close to that calculated by the finite element method, with minor differences only at the surface of the core layer. These differences have a minimal effect on the bending deformation of the structure, indicating that the theoretical model in this study can be used for the stress analysis of sandwich panels.

#### 5.1.2. Displacement Analysis

**Figure 11:**
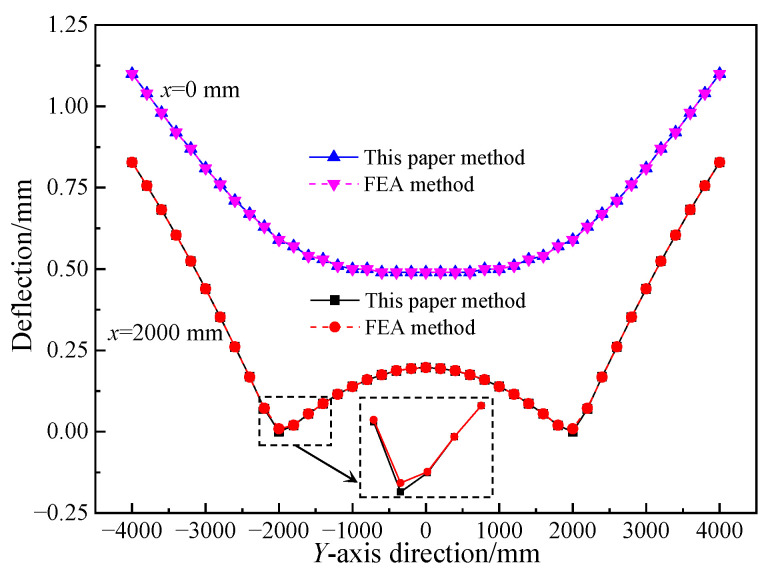
Comparison of sandwich panel calculation results.

Figure 11 shows the deflection of the middle plane of a concrete sandwich panel with four-point hinged support conditions at *c* = 2000 mm. From Figure 11, it can be observed that the vertical displacements of the middle plane of the sandwich panel at *x* = 0 mm calculated using the method proposed in this paper and the finite element method are in good agreement. However, there are some differences in the results between the two methods at the *x* = 2000 mm boundary, with a minor difference within a range of approximately 200 mm near the support positions. The analysis indicates that this is mainly due to the neglect of compression and shear deformations in the thickness direction of the sandwich panel in the proposed method. Considering the distribution of support reactions, it can be inferred that the error distribution area extremely small, and the error is relatively small. Since the vertical displacements on both sides of the support are small, and the simplified theoretical model can effectively improve computational efficiency, it demonstrates that the proposed calculation method is reasonable and feasible, and the numerical results are accurate.

### 5.2. Deformation Pattern of Sandwich Panels

**Figure 12:**
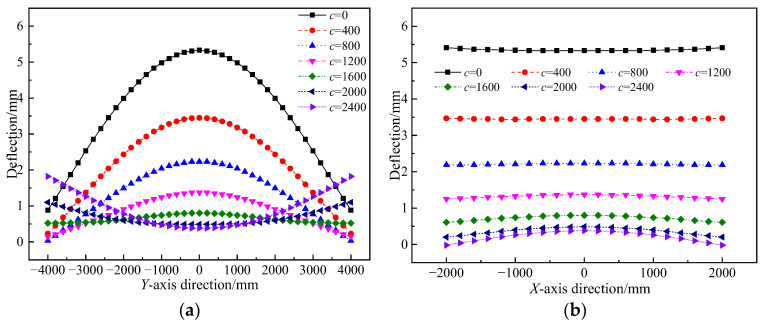
Bending deformation curve of a four-point supported sandwich panel: (**a**) *x* = 0 mm mid-plane line; and (**b**) *y* = 0 mm mid-plane line.

The variation in support positions notably influences the flexural deformation of key sections and characteristic points of concrete sandwich panels. Figure 12 illustrates the flexural deformation patterns of concrete sandwich panels at the longitudinal mid-span section (*x* = 0 mm) and the transverse mid-span section (*y* = 0 mm) under varied support positions.

As shown in Figure 12, the bending shapes of the longitudinal and transverse mid-span sections change with variations in support end distances. When the support end distance *c* ≤ 1600 mm, the longitudinal mid-span section bends upwards, while, when *c* > 1600 mm, the bending shape changes to downwards. For the transverse mid-span section, when *c* ≤ 1600 mm, the section remains mostly horizontal, and, when *c* > 1600 mm, it exhibits an upward bending shape. The analysis from Figure 12 reveals that, when the support span distance is between 1600 to 2000 mm, the overall deflection of the longitudinal and transverse mid-span sections of the concrete sandwich panels is relatively small. This indicates that, when the support is within this range, the load transfer efficiency of the sandwich panels is high, with a large structural stiffness and minimal deformation.

**Figure 13:**
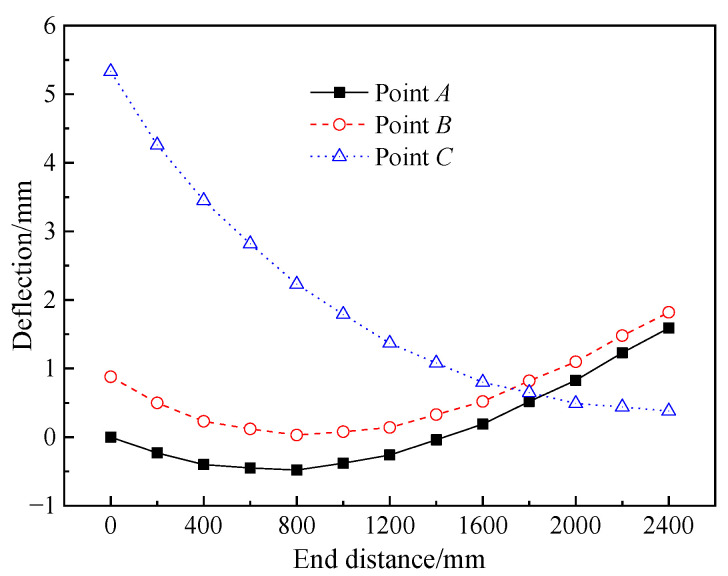
Vertical deflection variation curve of characteristic points.

Figure 13 shows the variation curve of the vertical deflection at characteristic points *A* (*x* = 2000 mm, *y* = 4000 mm), *B* (*x* = 0 mm, *y* = 4000 mm), and *C* (*x* = 0 mm, *y* = 0 mm) within the sandwich panel with changing support end distance *c*.

The analysis of Figure 13 demonstrates that, when the support end distance *c* = 0 mm, the sandwich panel’s point *C* exhibits the maximum vertical displacement of 8.77 mm. As the support end distance *c* increases, the vertical deflection of point *C* sharply decreases, while that of points *A* and *B* first decreases and then increases. The transition point for point *A* and point *B* are both at *c* = 800 mm, and, for point *B*, it is at *c* = 800 mm. Combining the deflection patterns of points *A*, *B*, and *C*, it is observed that, when the support end distance *c* = 1800 mm, the overall vertical displacement of the sandwich panel is minimized. At this transition point, the vertical deflection of each characteristic point is not greater than 0.80 mm. This is due to the movement of structural support points, which gradually disperses the plate load and shortens the load transfer path, thus enhancing the efficiency of load transfer on the plate surface. This conclusion is consistent with the displacement analysis results from Figure 12.

### 5.3. Bending Moment Analysis of Sandwich Panels

**Figure 14:**
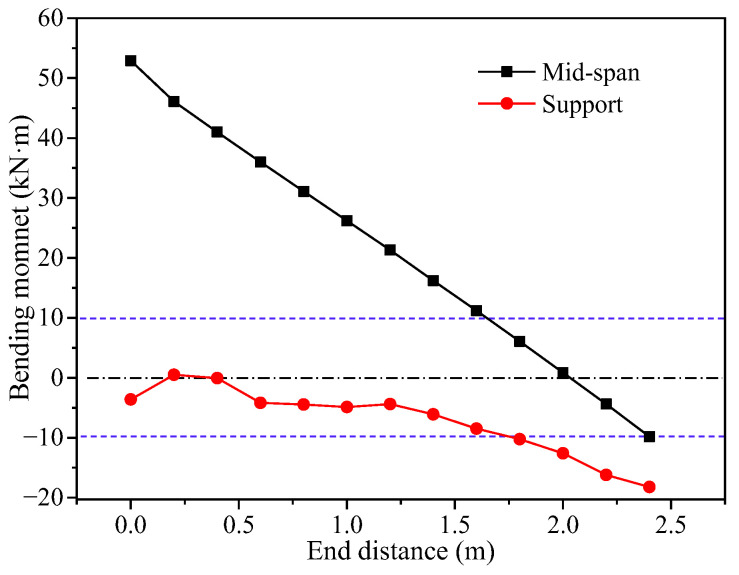
Law of bending moment for characteristic cross-section.

Under different loading conditions at various concentrated support points, the longitudinal mid-span bending moment (section at *y* = 0 mm) and support bending moment of concrete sandwich panel structures can reflect the main internal forces of the concrete sandwich structure. According to the calculation results in Figure 14, the curves showing the variations of the longitudinal mid-span bending moment and support bending moment with respect to the support end distance are presented.

Analyzing Figure 14, it can be observed that, as the support end distance increases, the mid-span length decreases, resulting in a decrease in the mid-span bending moment and a gradual increase in the negative moment at the support. Overall, the magnitude of the mid-span positive moment is greater than the negative moment at the support. According to the principle of equivalent strength design, using the blue dashed line in Figure 14 as the permissible line for bending moment forces (−10~10 kN·m), only the condition with *c* = 1800 mm satisfies the design requirements. This indicates that the concrete sandwich panel structure meets the design criteria when the support end distance is *c* = 1800 mm, resulting in the overall minimum bending moment and high structural load efficiency. This analytical conclusion aligns with the reasonable support end distance for the vertical deflection of concrete sandwich panels shown in Figure 13.

## 6. Conclusions

Based on Kirchhoff’s theory, this paper establishes a theoretical calculation model for concrete sandwich plate structures. The bending differential equations of elastic plates are derived and the accuracy of the calculation method is verified through a finite element simulation analysis. Additionally, the investigation examines the deformation and internal force variation laws of concrete sandwich panels under a four-point concentrated support boundary, leading to the following conclusions:

(1) When establishing the theoretical model of a concrete sandwich panel, it is necessary to consider the difference in concrete panel thickness and the mechanical properties of each layer of materials. The concrete wythe layer typically has a larger thickness and a higher elastic modulus, while the core layer is softer in texture with a lower elastic modulus. The significant differences in the mechanical properties between the surface layer and the core layer result in weak interactions at the interface, with the core layer bearing minimal structural internal forces. The bending normal stress in the concrete panel is unevenly distributed along the thickness direction, requiring a layered discussion when constructing the calculation model for sandwich plates.
(2) The optimal support positions for the four-point supported concrete sandwich panel is at support end distance 0.2*l* to 0.25*l*. When the distance between the concentrated hinged support points increases, the vertical deflection at the characteristic points of the concrete sandwich panel shows a consistent decrease or a trend of initial decrease followed by an increase, while the bending moment internal forces at the characteristic sections exhibit increasing or decreasing patterns, respectively. In the range of support end distances from *c* = 1600 mm to 2000 mm, the overall vertical deflection and bending moment internal forces of the concrete sandwich panel are relatively small, with a uniform stiffness distribution and high load transfer efficiency.
(3) The concrete sandwich panel calculation method established in this paper is simple and the results are accurate. The stress distribution obtained from this calculation method along the thickness direction of the panel is in basic agreement with the finite element calculation results, with a simpler stress distribution pattern. Additionally, the vertical displacement calculation results from this method compared to the finite element analysis show minimal differences, with errors only occurring in the localized support regions due to the neglect of compressive and shear deformations in the core layer. These errors are confined to a small region of the sandwich panel area. However, it is important to note that the application premise of this calculation method is that there are reliable connections between the concrete panels to ensure that they are jointly subjected to loads.

## Figures and Tables

**Table 1:** Research status of concrete sandwich panels.

Authors and References	Materials		Connector Type	Research Contents
	Wythe Layer	Core Layer		
Benayoune et al. [3]	RC	XPS	Reinforced truss	Compression
Wang et al. [4]	RC	XPS	Reinforced truss	Wallboard seismic
Gara et al. [5]	RC	XPS	Z-reinforced	Compression
Cheng et al. [6]	RC	XPS	Reinforced; Shaped steel	Large-span roof
Liu et al. [7]	RC	XPS	Z-reinforced (BFRP)	Wallboard flexural
Kumar et al. [8]	RC	XPS	Hollow GFRP tubes	Flexural
Zheng et al. [9]	RC	EPS	Concrete ribs	Flexural
Zhao et al. [10]	RC	EPS	Z-reinforced	Wallboard seismic
Hou et al. [11]	RC	EPS	Reinforced truss	Wallboard flexural
Xu et al. [12]	RC	/	Reinforced truss	Construction stage
Huang et al. [13]	RC	XPS, EPS	Reinforced truss	Temperature effect
Kim et al. [14]	RC	XPS, EPS	Grid-type GFRP	Flexural
Xun et al. [15]	TRC	Foam concrete	Fabric mesh	Flexural; Shear
Yang et al. [16]	TRC	EPS mortar	/	Flexural by impact
Fu [17]	Steel wire concrete	XPS	Concrete cylinder	Flexural
Joseph et al. [18]	Steel wire concrete	EPS	Steel wires truss	Flexural
Lameiras et al. [19]	Steel fiber concrete	XPS	Z-reinforced (GFRP)	Compression bending
Xie et al. [20]	RA concrete	XPS	Z-reinforced (BFRP)	Flexural
Li et al. [21]	Fine stone concrete	XPS	Steel wire mesh	Flexural
Mugahed et al. [22]	Foam concrete	XPS	Reinforced truss	Flexural
Umer et al. [23]	Glass fabric	PVC foam	/	Flexural
Zhou et al. [24]	CFRP	PVC foam	Z-reinforced	Vibration
Ding et al. [25]	UHPC	XPS	Short UHPC columns	Flexural
Zhang et al. [26]	GFRP	XPS	Rivets	Flexural

Note: Expanded Polystyrene (XPS), Reinforced Concrete (RC), Textile Reinforced concrete (TRC), Glass-Fiber-Reinforced Polymer (GFRP), Basalt-Fiber-Reinforced Polymer (BFRP), Ultra-High Performance Concrete (UHPC), Polyvinyl Chloride (PVC), Carbon-Fiber-Reinforced Plastics (CFRP), Recycled Aggregate (RA).

**Table 2:** Material physical mechanical index parameters.

Material Category	Volumetric Weight /kN·m^−3^	Elastic Modulus /N·mm^−2^	Poisson’s Ratio	Shear Modulus /N·mm^−2^
Concrete	25.00	3 × 10^4^	0.20	1.25 × 10^4^
Extruded Polystyrene	0.35	40	0.00	20
Reinforcement	78.50	2.06 × 10^5^	0.30	7.92 × 10^4^

## Data Availability

The data are contained within the article.
